# Intravenously administered cloxacillin-induced neutropenia with eosinophilia in a patient with infective endocarditis: a case report

**DOI:** 10.1186/s13256-018-1933-3

**Published:** 2018-12-29

**Authors:** J. A. A. S. Jayaweera, W. P. H. Abeydeera, G. R. Ranasinghe

**Affiliations:** 1 Department of Microbiology Faculty of Medicine and Allied Sciences, Rajarata University of Sri Lanka, Saliyapura, Sri Lanka; 2 Teaching Hospital Kurunegala, Kurunegala, Sri Lanka

**Keywords:** Infective endocarditis, Cloxacillin, Prolonged use, Agranulocytosis, Eosinophilia and risk for sepsis

## Abstract

**Background:**

Bacteremia following *Staphylococcus aureus* is a serious clinical condition which is often associated with distant metastatic infections. One of the most dreaded complications of *Staphylococcus aureus bacteremia* is infective endocarditis. Cloxacillin is a common antibiotic prescribed for suspected staphylococcal infections and confirmed methicillin-sensitive *Staphylococcus aureus* infections. Prolonged use of cloxacillin may lead to neutropenia.

**Case presentation:**

A 38-year-old Sinhalese man presented to Teaching Hospital Kurunegala, Sri Lanka, complaining of a 3-week history of fever; he was found to have a pansystolic murmur over the apex of his heart. He had leukocytosis with predominant neutrocytosis. His C-reactive protein was 231 mg/l and erythrocyte sedimentation rate was 100 mm/first hour. Transthoracic two-dimensional echocardiography revealed prolapsed mitral valve with 7 × 13 mm vegetation over the posterior mitral valve. On the following day, three blood cultures became positive and were subsequently identified as *Staphylococcus aureus*. Intravenously administered cloxacillin 3 g 6 hourly was started. Following day 24 of intravenously administered cloxacillin, our patient developed high spike fever. His total white blood cells were: 990/mm^3^ with 34% neutrophils and 22% eosinophils. His hemoglobin concentration was 9.5 g/dL and platelet count remained normal (202 × 10^6^/mm^3^). His C-reactive protein was 78 mg/l, erythrocyte sedimentation rate was 95 mm/first hour, and he was otherwise comfortable, showing no signs of sepsis beside the high grade fever. His serum was negative for filarial and *Toxoplasma* antibodies while stool was negative for oocytes and amoebic cysts. Further, his serum was negative for dengue virus, Epstein–Barr virus, cytomegalovirus, and hepatitis B antibodies. He was clinically well on day 6 after stopping cloxacillin with 44% neutrophils and 18% eosinophils. His C-reactive protein and erythrocyte sedimentation rate became normal, and there was no further plan for cardiothoracic intervention or administration of antimicrobials. He was discharged from hospital and remained well 6 months later.

**Conclusion:**

This case report signifies the potential fatal adverse effect of cloxacillin in methicillin-sensitive *Staphylococcus aureus* infections. Leukopenia is associated with prolonged use of high doses of cloxacillin. In addition to transthoracic two-dimensional echocardiography and inflammatory markers, sequential white blood cells and differential counts would help clinicians to assess the prognosis of patients with infective endocarditis.

## Background

Bacteremia following *Staphylococcus aureus* is a serious clinical condition which is often associated with distant metastatic infections [1, 2]. One of the most dreaded complications of *Staphylococcus aureus* bacteremia is infective endocarditis (IE), which has been reported to occur in 6–32% of these patients [3, 4].

Cloxacillin is one of several common antibiotics prescribed for suspected staphylococcal infections and confirmed methicillin-sensitive *Staphylococcus aureus* (MSSA) infections [5]. In IE, prolonged use of antimicrobials is advocated according to the guidelines of the Infectious Diseases Society of America; thus, it warrants complete cure of IE with minimal complications. However, prolonged (> 20 days) use of cloxacillin may lead to neutropenia [6]. In this case, prolonged use of cloxacillin in MSSA-associated IE led to neutropenia which is described here.

## Case presentation

A 38-year-old Sinhalese man presented to Teaching Hospital Kurunegala in Sri Lanka complaining of a 3-week history of fever, lethargy, and fatigability. He had not had any significant clinical conditions prior to this. He did not have any food or drug allergies while he was on regular anti-worm and anti-filarial prophylaxis. On clinical examination, he was febrile (39.4 °C), pale, and found to have a pansystolic murmur over the apex of his heart. He had leukocytosis (12.4 mm^3^) with predominant neutrocytosis (81%). His hemoglobin was 11.2 g/dL, C-reactive protein (CRP) was 231 mg/l, and erythrocyte sedimentation rate (ESR) was 100 mm/first hour. Transthoracic two-dimensional echocardiography (echo) revealed grade II mitral regurgitation, myxomatous, prolapsed mitral valve with 7 × 13 mm vegetation over the posterior mitral valve. Three sets of blood cultures were obtained within 1 hour from three different venipuncture sites; intravenously administered ceftriaxone was started empirically.

The following day, the three blood cultures became positive and were subsequently identified as *Staphylococcus aureus*. Intravenously administered cloxacillin 3 g 6 hourly was initiated while ceftriaxone was omitted. The (72 hours following initial culture) clearance blood cultures revealed *Staphylococcus aureus* and repeated clearance cultures following 72 hours remained negative. From that day onwards, for duration of 42 days, intravenously administered cloxacillin therapy was determined while clinical response was monitored with quarter hourly temperature, transthoracic two-dimensional echo, white blood cell/differential counts (WBC/DC), CRP, and ESR (Table 1). Further, he was on acetaminophen and chlorpheniramine malate as required.

**Table 1:** Timeline: fluctuation of hematological parameters, inflammatory markers, and transthoracic two-dimensional echocardiography following intravenously administered cloxacillin treatment

	Timeline								
Parameter	On IV cloxacillin therapy				Omission of IV cloxacillin				
Hematological	On admission	Day _12_	Day _17_	Day _22_	Day _24_	Day _26_	Day _30_	Day _32_	Day _34_
WBC/mm^3^	12.4	8.2	8.1	3.5	2.5	3.1	4.2	4.4	4.4
N (%)	81	70	65	59	34	41	44	40	50
E (%)	1	1	1	6	22	22	18	18	9
Hemoglobin (g/dL)	11.2	10.9	10.8	10.0	9.5	10.1	10.9	10.9	10.0
Platelets (10^6^/mm^3^)	219	430	400	293	202	208	281	302	290
Inflammatory									
CRP (mg/l)	231	56	29	16	78	102	68	32	8
ESR mm/first hour	100	95	88	68	95	90	78	70	56
Transthoracic two-dimensional echo									
Vegetation	Yes	Yes	Yes	Yes	No	No	No	No	No
Size (mm)	7 × 13	7 × 10	6 × 10	4 × 8	–	–	–	–	–

*CRP* C-reactive protein, *E* eosinophils, *echo* echocardiography, *ESR* erythrocyte sedimentation rate, *IV* intravenously administered, *N* neutrophils, *WBC* white blood cells

Following day 24 of intravenously administered cloxacillin, our patient developed high spike fever (39.6 °C) and his full blood count showed: WBC 990/mm^3^ with 34% of neutrophils and 22% eosinophils. His hemoglobin concentration was 9.5 g/dL, platelet count (202 × 10^6^/mm^3^), D-dimer (320 ng/mL fibrinogen equivalent units), and both prothrombin time (PT) and partial thromboplastin time (PTT) remained normal. His CRP was 78 mg/l, ESR was 95 mm/first hour, and he was otherwise comfortable, showing no signs of sepsis beside the high grade fever. His vital signs (blood pressure and pulse) were normal. Repeat transthoracic two-dimensional echo was normal thus no vegetations were detected. Mild elevation of liver enzymes was observed and an ultrasound of his abdomen revealed no hepatomegaly: gamma-glutamyl transferase 192 IU/ml, alanine transaminase (ALT) 15 IU/ml, and alkaline phosphatase (ALP) 136 IU/ml. We omitted intravenously administered cloxacillin and kept him without antimicrobials while arranging a septic screening with a close observation of clinical parameters, WBC/DC, and inflammatory markers. His blood picture showed leukopenia with profound neutropenia and he had eosinophilia. Red blood cells and platelets were normal. He was clinically well and on day 6 after stopping cloxacillin, white blood cells (WBC) became normal with 44% neutrophils and 18% eosinophils. Liver function tests also returned to normal after cloxacillin discontinuation. He was treated with anti-pyretic as required. Subsequently, his septic screening became negative and transthoracic two-dimensional echo showed complete healing with no vegetations.

His serum was negative for filarial and *Toxoplasma* antibodies while stool was negative for oocytes and amoebic cysts. Further, his serum was negative for Epstein–Barr virus, cytomegalovirus, and hepatitis B antibodies. Subsequently his CRP and ESR became normal, and there was no further plan for cardiothoracic intervention or administration of antimicrobials. He was discharged from hospital and remained well 6 months later.

## Discussion

IE if left untreated is almost inevitably fatal. Prognosis mainly depends on whether or not complications develop. Early detection and appropriate treatment of this uncommon disease can be lifesaving. The overall mortality rate has remained stable at 14.5%. To eradicate the bacteria that remain inside the vegetation in IE requires prolonged and high-dose antimicrobial treatment. For community-acquired MSSA infection in individuals who do not abuse intravenously administered drugs, the cure rate is 60–70% [7].

Neutrophils are a type of immune cell, termed granulocytes, and they are the first cell types to travel to the site of an infection. Neutrophils help fight infection by ingesting microorganisms and releasing enzymes that kill the microorganisms. Agranulocytosis is a marked and profound decrease in the number of granulocytes, or an absolute lack of granulocytes in circulating blood, typically resulting in a neutrophil count below 0.5 × 10^9^/L [8]. Most cases (70–90%) of agranulocytosis are caused by viral infections, drugs (drug or its metabolites), chemotherapy, and radiotherapy [9]. Beta lactams and especially penicillins have been reported to cause agranulocytosis since 1946 [10], and have long been associated with the inhibition of granulopoiesis. A few antibiotics other than beta lactam, such as linezolid, trimethoprim-sulfamethoxazole, clindamycin, gentamycin, and chloramphenicol, are known to cause agranulocytosis [11]. Simultaneously, development of eosinophilia signifies parasitic infestation or development of hypersensitivity or both.

In this case scenario, we had a fair amount of doubt about the definitive cause of the emergence of pyrexia and the hematological discrepancy. Top of the list was MSSA relapse, new infection with methicillin-resistant *Staphylococcus aureus* (MRSA) or different Gram-negative bacteria or viral agent, or parasitic infestation leading to pyrexia with leukopenia and eosinophilia. Often, isolated bacterial, viral, or parasitic infections do not produce a combined picture of neutropenia with eosinophilia. In parasitic infestation, eosinophilia can develop without neutropenia, whereas in viral infections, neutropenia can develop without eosinophilia [12]. Co-infection of viral and parasitic agent can lead to the above picture [13]. However, we excluded common viral infections and parasitic infestations which are endemic in Sri Lanka. A repeat transthoracic two-dimensional echo was normal and excluded the possibility of valvular or myocardial abscesses. Also, his D-dimer, PTT, and PT were normal; the possibility of disseminated intravascular coagulation was excluded.

Following omission of cloxacillin and our patient’s significant recovery with subsiding fever and normalizing of his hematological parameters, our attention was directed toward adverse drug reactions. The impact of this case report is that it signifies the potential fatal adverse effect of a commonly used antimicrobial in MSSA infections: cloxacillin. Often this phenomenon is associated with prolonged use of high doses. A case report in 2014 described the use of cloxacillin in a patient with MSSA-IE and agranulocytosis developed in 20 days of therapy [6]. In our case it was after 24 days and the total dose was 288 g of cloxacillin. It was postulated that total dose of > 150 g was associated with the development of neutropenia [6]. Our patient was on intravenously administered ceftriaxone as well but it was given over 2 days thus the effect could be negligible.

The exact pathogenesis behind the leukopenia was less understood and two mechanisms were proposed: direct toxic effect of the drug, and immunological allergic reaction and creation of neutrophil antibodies. This patient developed leukopenia associated with neutropenia while hemoglobin and platelets remained normal. This reflects suppression only in granulocyte axis of cell genesis. Also, during the leukopenia phase our patient had eosinophilia which suggests immune-mediated hypersensitivity [6, 14].

Although this is a rare phenomenon, awareness among clinicians of such adverse reactions would alert them to assess the patient periodically with serial WBC/DC. In the documented cases, including this case, the patients only developed high grade fever with high CRP and did not develop neutropenic sepsis [6]. Also, once cloxacillin was omitted WBC/DC became normal. The prolonged use of cloxacillin with high dose in clinical practice is quite common, but the occurrence of agranulocytosis is quite rare. For development of agranulocytosis there could be an existing genetic predisposition for the development of cloxacillin-induced hypersensitivity or drug-induced myelotoxicity.

In IE, prolonged (28–42 days) intravenously administered antibiotics are required to eradicate the microbes within the cardiac vegetation. Here, following early and appropriate therapy, our patient became afebrile and clinically stable. When febrile neutropenia develops, clinicians often tend to prescribe granulocyte colony-stimulation factor (GCSF) to stimulate granulocyte formation. This was practiced commonly among patients with cancer with IE. Here, however, the probable cause of neutropenia was drug induced and therefore we omitted the offending agent and did not give GCSF [9, 15].

## Conclusion

Prolonged administration of cloxacillin with high doses could lead to the development of leukopenia which puts patients at risk of developing neutropenic sepsis. Vigilant attention on blood WBC/DC would help clinicians to identify the leukopenia early and withdraw the cloxacillin. In addition to transthoracic two-dimensional echo, CRP, ESR, and sequential WBC/DC would help clinicians to assess the prognosis of patients with IE.

## Abbreviations

ALP: Alkaline phosphatase
ALT: Alanine transaminase
CRP: C-reactive protein
Echo: Echocardiography
ESR: Erythrocyte sedimentation rate
GCSF: Granulocyte colony-stimulation factor
IE: Infective endocarditis
MRSA: Methicillin-resistant *Staphylococcus aureus*
MSSA: Methicillin-sensitive *Staphylococcus aureus*
PT: Prothrombin time
PTT: Partial thromboplastin time
WBC: White blood cells
WBC/DC: White blood cell/differential counts

## References

[1] Eggimann P, Pittet D. Acute bacteraemia. In: Fink MP, Abraham E, Vincent J, Kochanek P, editors. Textbook of critical care. Philadelphia: Elsevier Saunders; 2005. p. 1275–83.

[2] Lyytikainen O, Ruotsalainen E, Jarvinen A, Valtonen V, Ruutu P. Trends and outcome of nosocomial and community-acquired bloodstream infections due to *Staphylococcus aureus* in Finland, 1995–2001. Eur J Clin Microbiol Infect Dis. 2005;24:399–404. 10.1007/s10096-005-1345-3 15931453

[3] Benfield T, Espersen F, Frimodt-Moller N, Jensen AG, Larsen AR, Pallesen LV, *et al.* Increasing incidence but decreasing in-hospital mortality of adult *Staphylococcus aureus* bacteraemia between 1981 and 2000. Clin Microbiol Infect. 2007;13:257–63. 10.1111/j.1469-0691.2006.01589.x 17391379

[4] Rieg S, Peyerl-Hoffmann G, de With K, Theilacker C, Wagner D, Hubner J, *et al.* Mortality of *S. aureus* bacteremia and infectious diseases specialist consultation—a study of 521 patients in Germany. J Inf Secur. 2009;59:232–9. 10.1016/j.jinf.2009.07.015 19654021

[5] Wijesekara PNK, Kumbukgolla WW, Jayaweera JAAS, Rawat D. Review on Usage of Vancomycin in Livestock and Humans: Maintaining Its Efficacy, Prevention of Resistance and Alternative Therapy. Vet Sci. 2017;4:6. 10.3390/vetsci4010006 PMC5606620 29056665

[6] Wong KW. Intravenous Cloxacillin Induced Agranulocytosis in a Haemodialysis Patient. Gen Med (Los Angel). 2014;2:142. 10.4172/2327-5146.1000142.

[7] Bor DH, Woolhandler S, Nardin R, Brusch J, Himmelstein DU. Infective endocarditis in the USA 1998-2009: a nationwide study. PLoS One. 2013;8(3):e60033. 10.1371/journal.pone.0060033 PMC3603929 23527296

[8] Andrès E, Zimmer J, Affenberger S, Federici L, Alt M, *et al.* Idiosyncratic drug-induced agranulocytosis: Update of an old disorder. Eur J Intern Med. 2006;17:529–35. 10.1016/j.ejim.2006.07.012 17142169

[9] Andrès E, Zimmer J, Mecili M, Weitten T, Alt M, *et al.* Clinical presentation and management of drug-induced agranulocytosis. Expert Rev Hematol. 2011;4:143–51. 10.1586/ehm.11.12 21495924

[10] Spain DM, Clark TB. A case of agranulocytosis occurring during the course of penicillin therapy. Ann Intern Med. 1946;25:732. 10.7326/0003-4819-25-4-732 20999605

[11] Apinantriyo B, Lekhakula A, Rujirojindakul P. Incidence, etiology and bone marrow characteristics of non-chemotherapy-induced agranulocytosis. Hematology. 2011;16:50–3. 10.1179/102453311X12902908411715 21269568

[12] Weatherall DJ, Kwiatkowski D. Hematologic manifestations of systemic diseases in children of the developing world. In: Nathan D, Orkin, editors. Hematology of Infancy and Childhood. 5th ed. Philadelphia: WB Saunders; 2012. p. 1902.

[13] Esposito DH, Stich A, Epelboin L, *et al.* Acute muscular sarcocystosis: an international investigation among ill travelers returning from Tioman Island, Malaysia, 2011-2012. Clin Infect Dis. 2014;59:1401. 10.1093/cid/ciu622 PMC4624310 25091309

[14] Tanous O, Hayek T, Hamoud S. Transient Cloxacillin Induced granulocytosis with Eosinophilia and Elevated IgE Levels. Gen Med (Los Angel). 2013;1:111.

[15] Borgbjerg BM, Hovgaard D, Laursen JB, Aldershvile J. Granulocyte colony stimulating factor in neutropenic patients with infective endocarditis. Heart. 1998;79(1):93–5. 10.1136/hrt.79.1.93 PMC1728566 9505928

